# New Proofs of Some *q*-Summation and *q*-Transformation Formulas

**DOI:** 10.1155/2014/940358

**Published:** 2014-05-07

**Authors:** Xian-Fang Liu, Ya-Qing Bi, Qiu-Ming Luo

**Affiliations:** Department of Mathematics, Chongqing Higher Education Mega Center, Chongqing Normal University, Huxi Campus, Chongqing 401331, China

## Abstract

We obtain an expectation formula and give the probabilistic proofs of some summation and transformation formulas of *q*-series based on our expectation formula. Although these formulas in themselves are not the probability results, the proofs given are based on probabilistic concepts.

## 1. Introduction

The probabilistic method is an important tool to derive results in combinatorics, theory of numbers, and other fields (see [1–15]). There have been many applications in the basic hypergeometric series (or *q*-series). For example, Fulman [3] presented a probabilistic proof of Rogers-Ramanujan identity using Markov chain. Chapman [2] proved the Andrews-Gordon identity by using extended Fulman's methods. Kadell [4] gave a probabilistic proof of Ramanujan's _1_ *ψ*_1_ summation based on the order statistics.

Recently, Wang [13, 14] constructed a random variable *X* and introduced a new probability distribution *W*(*x*; *q*):

(1) P(X=xnqk)=pn,k(x;q)=∶(−x)nqk(xn−1qk+1,xnqk+1;q)∞(q,q/x,x;q)∞,

where

(2) pn,k(x;q)>0, ∑pn,k(x;q)=1,x<0,  0<q<1,  n=0,1,  k=0,1,2,….

By applying the above probability distribution, Wang proved the *q*-binomial theorem and *q*-Gauss summation formula and also obtained some new summation formulas and transformation formulas.

One of the most important concepts in probability theory is that of the expectation of a random variable. If *X* is a discrete random variable having a probability mass function *p*(*x*), then the* expectation*, or the* expected value*, or the* expectation operator* of *X*, denoted by *E*[*X*], is defined by (e.g., [9, page 125])

(3) $$\begin{array}{c} E\left[X\right]=\overset{}{\underset{p\left(x\right)>0}{\sum }}xp\left(x\right). \end{array}$$

In the following section we introduce some notations, definitions, and formulas of *q*-series. Throughout this paper we suppose *q* ∈ *C*, |*q*| < 1.

The *q*-shifted factorials are defined by

(4) $$\begin{array}{c} {\left(a;q\right)}_{0}=1,\;{\left(a;q\right)}_{n}=\overset{n-1}{\underset{k=0}{\prod }}\left(1-aq^{k}\right),\\ {\left(a;q\right)}_{\infty }=\overset{\infty }{\underset{k=0}{\prod }}\left(1-aq^{k}\right),\;n\geq 1. \end{array}$$

Clearly,

(5) $$\begin{array}{c} {\left(a;q\right)}_{n}=\frac{{\left(a;q\right)}_{\infty }}{{\left(aq^{n};q\right)}_{\infty }},\\ {\left(a^{2};q^{2}\right)}_{n}={\left(a;q\right)}_{n}{\left(-a;q\right)}_{n}. \end{array}$$

The following are compact notations for the multiple *q*-shifted factorials:

(6) $$\begin{array}{c} {\left(a_{1},a_{2},\ldots ,a_{m};q\right)}_{n}={\left(a_{1};q\right)}_{n}{\left(a_{2};q\right)}_{n}\cdots {\left(a_{m};q\right)}_{n},\\ {\left(a_{1},a_{2},\ldots ,a_{m};q\right)}_{\infty }={\left(a_{1};q\right)}_{\infty }{\left(a_{2};q\right)}_{\infty }\cdots {\left(a_{m};q\right)}_{\infty }. \end{array}$$

The basic hypergeometric series or *q*-series _*r*_ *ϕ*_*s*_ are defined by (see [16, 17])

(7) ϕsr(a1,a2,…,arb1,b2,…,bs;q,z)  =∑n=0∞(a1,a2,…,ar;q)n(q,b1,b2,…,bs;q)n[(−1)nq(n2)]1+s−rzn.

Heine introduced the _*r* + 1_ *ϕ*_*r*_ basic hypergeometric series which is defined by

(8) ϕrr+1(a1,a2,…,ar+1b1,b2,…,br;q,z)=∑n=0∞(a1,a2,…,ar;q)n(q,b1,b2,…,br;q)nzn.

Jackson defined the *q*-integral (see [17, 18]):

(9) $$\begin{array}{c} \overset{d}{\underset{0}{\int }}f\left(t\right)d_{q}t=d\left(1-q\right)\overset{\infty }{\underset{n=0}{\sum }}f\left(dq^{n}\right)q^{n}, \end{array}$$

(10) $$\begin{array}{c} \overset{d}{\underset{c}{\int }}f\left(t\right)d_{q}t=\overset{d}{\underset{0}{\int }}f\left(t\right)d_{q}t-\overset{c}{\underset{0}{\int }}f\left(t\right)d_{q}t. \end{array}$$

The following is the Andrews-Askey integral (see [19]) which can be derived from Ramanujan's _1_ *ψ*_1_:

(11) $$\begin{array}{c} \overset{d}{\underset{c}{\int }}\frac{{\left(qt/c,qt/d;q\right)}_{\infty }}{{\left(at,bt;q\right)}_{\infty }}d_{q}t=\frac{d\left(1-q\right){\left(q,dq/c,c/d,abcd;q\right)}_{\infty }}{{\left(ac,ad,bc,bd;q\right)}_{\infty }}, \end{array}$$

provided that there are no zero factors in the denominator of the integrals. Recently, Liu and Luo [20] further generalized the above Andrews-Askey integral in the following more general form.

**Lemma 1 (see [21, page 5. (2.5)] [20, Theorem 1])**

One has

(12) ∫ab(qt/a,qt/b,ct;q)∞(et,ft,ht;q)∞dqt  =b(1−q)(q,bq/a,a/b,bc,abef;q)∞(ae,af,be,bf,bh;q)∞ϕ23   ×(be,bf,chabef,bc;q,ah),

provided |*ah*| < 1 and |*q*| < 1, provided that there are no zero factors in the denominator of the integrals.

**Lemma 2 (see [21, page 5. (2.7)])**

One has

(13) ∫ab(qt/a,qt/b,ct;q)∞(et,ht;q)∞dqt  =b(1−q)(q,bq/a,a/b,bc;q)∞(ae,be,bh;q)∞ϕ12(be,chbc;q,ah),

provided |*ah*| < 1 and |*q*| < 1, provided that there are no zero factors in the denominator of the integrals.

The aim of the present paper is to give an expectation formula and introduce some probabilistic proofs of the corresponding summation and transformation formulas of *q*-series based on an expectation formula. In Section 2 we give an expectation formula of the random variables (*dX*;*q*)_*∞*_/(*aX*,*bX*,*cX*;*q*)_*∞*_. In Section 3 we show the probabilistic proofs of transformation formulas of _3_ *ϕ*_2_. In Section 4 we give probabilistic proof of Heine's transformations and Jackson's transformations. In Section 5 we give probabilistic proof of some formulas of *q*-series, for example, *q*-binomial theorem, *q*-Chu-Vandermonde sum formulas, *q*-Gauss sum formula, *q*-Kummer sum formula, Bailey sum formula, and so forth.

## 2. Main Theorem

In this section we obtain the expectation formulas of some random variables which are very useful to prove the summation and transformation formulas of *q*-series.

**Theorem 3**

Let *X* denote a random variable with probability distribution *W*(*x*; *q*), −1 < *x* < 0. Then one has

(14) E[(dX;q)∞(aX,bX,cX;q)∞]  =(d,abx;q)∞(a,ax,b,bx,c;q)∞ϕ23(a,b,dcabx,d;q,cx),

provided that max⁡(|*a*|, |*b*|, |*c*|, |*d*|) < 1, |*cx*| < 1, and |*q*| < 1.

**Proof**

A random variable *X* has the distribution *W*(*x*; *q*). From definitions (9) we have

(15) ∫01(qt/x,qt,dt;q)∞(at,bt,ct;q)∞dqt  =(1−q)∑k=0∞(qk+1/x,qk+1,dqk;q)∞qk(aqk,bqk,cqk;q)∞,∫0x(qt/x,qt,dt;q)∞(at,bt,ct;q)∞dqt  =x(1−q)∑k=0∞(qk+1,xqk+1,dxqk;q)∞qk(axqk,bxqk,cxqk;q)∞.

From definitions (10) and combining (15) we have

(16) ∫x1(qt/x,qt,dt;q)∞(at,bt,ct;q)∞dqt  =(1−q)∑k=0∞(qk+1/x,qk+1,dqk;q)∞qk(aqk,bqk,cqk;q)∞   −x(1−q)∑k=0∞(qk+1,xqk+1,dxqk;q)∞qk(axqk,bxqk,cxqk;q)∞.

By using the probability distribution *W*(*x*; *q*) and noting (16) and (12) of Lemma 1, we calculate the expectation of the random variable (*dX*;*q*)_*∞*_/(*aX*,*bX*,*cX*;*q*)_*∞*_ as follows:

(17) E[(dX;q)∞(aX,bX,cX;q)∞]=∑n=01 ∑k=0∞(−x)n(xn−1qk+1,xnqk+1;q)∞qk(q,q/x,x;q)∞×(dxnqk;q)∞(axnqk,bxnqk,cxnqk;q)∞=1(1−q)(q,q/x,x;q)∞ ×((1−q)∑k=0∞(qk+1/x,qk+1,dqk;q)∞qk(aqk,bqk,cqk;q)∞−x(1−q)∑k=0∞(qk+1,xqk+1,dxqk;q)∞qk(axqk,bxqk,cxqk;q)∞)=1(1−q)(q,q/x,x;q)∞∫x1(qt/x,qt,dt;q)∞(at,bt,ct;q)∞dqt=1(1−q)(q,q/x,x;q)∞(1−q)(q,q/x,x,d,abx;q)∞(ax,bx,a,b,c;q)∞ϕ23. ×(a,b,dcabx,d;q,cx).

Hence, we obtain

(18) E[(dX;q)∞(aX,bX,cX;q)∞]  =(d,abx;q)∞(ax,a,bx,b,c;q)∞ϕ23(a,b,dcabx,d;q,cx).

The proof is complete.

**Theorem 4**

Let *X* denote a random variable with probability distribution *W*(*x*; *q*), −1 < *x* < 0. Then one has

(19) E[(dX;q)∞(aX,bX;q)∞]=(d,abx;q)∞(a,ax,b,bx;q)∞ϕ22(a,babx,d;q,dx),

provided that max⁡(|*a*|, |*b*|, |*d*|) < 1, |*dx*| < 1, and |*q*| < 1.

**Proof**

By (7) and (8) we have

(20) ϕ23(a,b,dcabx,d;q,cx)  =∑n=0∞(a;q)n(b;q)n(d/c;q)n(cx)n(q;q)n(abx;q)n(d;q)n  =∑n=0∞(a;q)n(b;q)nxn(q;q)n(abx;q)n(d;q)n(dc;q)ncn.

By (4) we have

(21) (dc;q)ncn=(1−dc)(1−dcq)⋯(1−dcqn−1)cn=(c−d)(c−dq)⋯(c−dqn−1).

Substituting (20) and (21) into the right-hand side of (14), we obtain

(22) E[(dX;q)∞(aX,bX,cX;q)∞] =(d,abx;q)∞(a,ax,b,bx,c;q)∞∑n=0∞(a;q)n(b;q)nxn(q;q)n(abx;q)n(d;q)n  ×(c−d)(c−dq)⋯(c−dqn−1).

Next, let us replace *c* by *λc*, respectively, and let *λ* → 0 in (22); we get

(23) E[(dX;q)∞(aX,bX;q)∞]=(d,abx;q)∞(a,ax,b,bx;q)∞∑n=0∞(a;q)n(b;q)nxn(q;q)n(abx;q)n(d;q)n ×(−d)(−dq)⋯(−dqn−1)=(d,abx;q)∞(a,ax,b,bx;q)∞∑n=0∞(a;q)n(b;q)nxn(q;q)n(abx;q)n(d;q)n ×dn(−1)nqn(n−1)/2=(d,abx;q)∞(a,ax,b,bx;q)∞∑n=0∞(a;q)n(b;q)n(dx)n(q;q)n(abx;q)n(d;q)n(−1)nq(n2)=(d,abx;q)∞(a,ax,b,bx;q)∞ϕ22(a,babx,d;q,dx).

The proof is complete.

**Theorem 5**

Let *X* denote a random variable with probability distribution *W*(*x*; *q*), −1 < *x* < 0. Then one has

(24) E[(cxX;q)∞(aX,bX,cX;q)∞] =(cx,abx;q)∞(ax,bx,a,b,c;q)∞ϕ23(a,b,xabx,cx;q,cx),

provided that max⁡(|*a*|, |*b*|, |*c*|) < 1, |*cx*| < 1, and |*q*| < 1.

**Proof**

Letting *d* = *cx* in (14) of Theorem 3, we obtain (24).

**Corollary 6 (see [13, page 463, Theorem 1])**

Let *X* denote a random variable with probability distribution *W*(*x*; *q*), −1 < *x* < 0. Then one has

(25) E[(cxX;q)∞(aX,bX,cX;q)∞] =(acx,bcx,x;q)∞(a,ax,b,bx,c,cx;q)∞ϕ23(ab,c,cxacx,bcx;q,x),

provided that max⁡(|*a*|, |*b*|, |*c*|) < 1.

**Proof**

Using (24) of Theorem 5, we deduce

(26) E[(cxX;q)∞(aX,bX,cX;q)∞] =(cx,abx;q)∞(ax,bx,a,b,c;q)∞ϕ23(a,b,xabx,cx;q,cx).

Using (31) of Theorem 8, we have

(27) ϕ23(a,b,xabx,cx;q,cx) =ϕ23(a,x,babx,cx;q,cx) =(x,bcx,acx;q)∞(abx,cx,cx;q)∞ϕ23(ab,c,cxbcx,acx;q,x).

Substituting (27) into the right-hand sides of (26), we have

(28) E[(cxX;q)∞(aX,bX,cX;q)∞]  =(cx,abx;q)∞(ax,bx,a,b,c;q)∞(x,bcx,acx;q)∞(abx,cx,cx;q)∞ϕ23   ×(ab,c,cxbcx,acx;q,x)  =(acx,bcx,x;q)∞(a,ax,b,bx,c,cx;q)∞ϕ23(ab,c,cxacx,bcx;q,x).

The proof is complete.

**Corollary 7 (see [14, page 245, Lemma 2.4])**

Let *X* denote a random variable with probability distribution *W*(*x*; *q*), −1 < *x* < 0. Then one has

(29) $$\begin{array}{c} E\left[\frac{1}{{\left(aX,bX;q\right)}_{\infty }}\right]=\frac{{\left(abx;q\right)}_{\infty }}{{\left(ax,bx,a,b;q\right)}_{\infty }}, \end{array}$$

provided that max⁡(|*a*|, |*b*|) < 1.

**Proof**

Letting *d* = *c* or *d* = *a* = *c* in (14) of Theorem 3, then we have

(30) E[1(aX,bX;q)∞]=(abx;q)∞(ax,bx,a,b;q)∞ϕ23(a,b,1abx,d;q,cx)=(abx;q)∞(ax,bx,a,b;q)∞.

The proof is complete.

## 3. Probabilistic Proofs of Transformation Formulas of _3_ *ϕ*_2_

Sears' _3_ *ϕ*_2_ transformation formula is widely applied to the special functions. In this section we will introduce probabilistic proofs of transformation of _3_ *ϕ*_2_.

**Theorem 8 (see [17, page 359. III. 9, III. 10])**

One has

(31) ϕ23(a,b,cd,e;q,deabc)  =(e/a,de/bc;q)∞(e,de/abc;q)∞ϕ23(a,db,dcd,debc;q,ea)

(32)    =(b,de/ab,de/bc;q)∞(d,e,de/abc;q)∞ϕ23(db,eb,deabcdeab,debc;q,b).

**Proof**

Interchanging *b* and *c* in (14), then we have

(33) E[(dX;q)∞(aX,bX,cX;q)∞] =(d,acx;q)∞(ax,a,cx,c,b;q)∞ϕ23(a,c,dbacx,d;q,bx).

Interchanging *a* and *c* in (14), then we have

(34) E[(dX;q)∞(aX,bX,cX;q)∞] =(d,bcx;q)∞(bx,b,cx,c,a;q)∞ϕ23(c,b,dabcx,d;q,ax).

By (14) and (33), we obtain

(35) ϕ23(a,b,dcabx,d;q,cx)  =(bx,acx;q)∞(cx,abx;q)∞ϕ23(a,c,dbacx,d;q,bx),

and, replacing (*a*, *b*, *d*/*c*, *d*, *abx*) by (*a*, *b*, *c*, *d*, *e*) in (35), we obtain a _3_ *ϕ*_2_ transformation formula

(36) ϕ23(a,b,cd,e;q,deabc)  =(e/a,de/bc;q)∞(e,de/abc;q)∞ϕ23(a,db,dcd,debc;q,ea).

By (14) and (34) and then replacing (*b*, *a*, *d*/*c*, *d*, *abx*) by (*a*, *b*, *c*, *d*, *e*), we obtain (31).

By (33) and (34), we have

(37) ϕ23(c,b,dabcx,d;q,ax)  =(bx,acx;q)∞(ax,bcx;q)∞ϕ23(a,c,dbacx,d;q,bx),

and, replacing (*c*, *b*, *d*/*a*, *d*, *b* *cx*) by (*a*, *b*, *c*, *d*, *e*) in (37), we obtain (32). The proof is complete.

## 4. Probabilistic Proof of Heine and Jackson's Transformations

Heine [22] derived transformation formulas for _2_ *ϕ*_1_ and also proved Euler's transformation formula. A basic hypergeometric representation for a given function is by no means unique. There are groups of transformation between various hypergeometric representations of the same function. We will first prove the classical Heine's transformation formula which will be useful in proving many other formulas. In this section we give the probabilistic proofs of Heine and Jackson's transformations.

**Theorem 9 (see [17, page 359, III. 1, III. 2, III. 3])**

Heine's transformation formulas for _2_ *ϕ*_1_ are

(38) ϕ12(a,bc;q,z)=(b,az;q)∞(c,z;q)∞ϕ12(cb,zaz;q,b)

(39) =(c/b,bz;q)∞(c,z;q)∞ϕ12(abzc,bbz;q,cb)

(40) =(abz/c;q)∞(z;q)∞ϕ12(ca,cbc;q,abzc).

**Proof**

Comparing (24) of Theorem 5 and (25) of Corollary 6, we obtain

(41) (acx,bcx,x;q)∞(a,ax,b,bx,c,cx;q)∞ϕ23(ab,c,cxacx,bcx;q,x) =(cx,abx;q)∞(ax,bx,a,b,c;q)∞ϕ23(a,b,xabx,cx;q,cx),

or, equivalently, that

(42) ϕ23(ab,c,cxacx,bcx;q,x) =(cx,abx,cx;q)∞(acx,bcx,x;q)∞ϕ23(a,b,xabx,cx;q,cx).

Setting *b* = 0 in (42), we have

(43) ϕ12(c,cxacx;q,x)=(cx,cx;q)∞(acx,x;q)∞ϕ12(a,xcx;q,cx).

Replacing (*c*, *cx*, *a* *cx*, *x*) by (*a*, *b*, *c*, *z*) in (43), we get

(44) ϕ12(a,bc;q,z)=(b,az;q)∞(c,z;q)∞ϕ12(cb,zaz;q,b)for  |b|<1,  |z|<1,

which is just (38).

Setting *d* = 0 and *a* = *b* and replacing *c* by *b* in (14), we have

(45) E[1(aX,aX,bX;q)∞]  =(a2x;q)∞(ax,ax,a,a,b;q)∞ϕ12(a,aa2x;q,bx).

Setting *d* = 0 and *a* = *c* in (14) of Theorem 3, we have

(46) E[1(aX,aX,bX;q)∞]  =(abx;q)∞(ax,bx,a,a,b;q)∞ϕ12(a,babx;q,ax).

Comparing (45) and (46), we obtain

(47) ϕ12(b,aabx;q,ax)=(bx,a2x;q)∞(abx,ax;q)∞ϕ12(a,aa2x;q,bx).

Replacing (*a*, *b*, *x*) by (*b*, *a*, *z*/*b*), we get

(48) ϕ12(a,baz;q,z)=(az/b,bz;q)∞(az,z;q)∞ϕ12(b,bbz;q,azb).

Letting *c* = *az* in (48) gives

(49) ϕ12(a,bc;q,z)=(c/b,bz;q)∞(c,z;q)∞ϕ12(abzc,bbz;q,cb).

We get (39). From (39) we can deduce (40).

Jackson's transformations formula is an important formula in basic hypergeometric series, and now we give a probabilistic proof of Jackson's transformation formulas for _2_ *ϕ*_1_ and _2_ *ϕ*_2_.

**Theorem 10 (see [17, page 359, III.4])**

Jackson's transformations of _2_ *ϕ*_1_, _2_ *ϕ*_2_ series are

(50) ϕ12(a,bc;q,z)=(az;q)∞(z;q)∞ϕ22(a,cbc,az;q,bz).

**Proof**

This includes employing two different forms of *E*[(*dX*;*q*)_*∞*_/(*aX*,*bX*;*q*)_*∞*_].

Letting *b* = 0 in (14) of Theorem 3 and then replacing *c* by *b*, we get

(51) E[(dX;q)∞(aX,bX;q)∞]=(d;q)∞(a,ax,b;q)∞ϕ12(a,dbd;q,bx).

Comparing (51) and (19) of Theorem 4 gives

(52) (d;q)∞(a,ax,b;q)∞ϕ12(a,dbd;q,bx)  =(d,abx;q)∞(a,ax,b,bx;q)∞ϕ22(a,babx,d;q,dx).

Then we obtain

(53) ϕ12(a,dbd;q,bx)=(abx;q)∞(bx;q)∞ϕ22(a,babx,d;q,dx).

Replacing (*a*, *d*/*b*, *d*, *bx*) by (*a*, *b*, *c*, *z*) gives

(54) ϕ12(a,bc;q,z)=(az;q)∞(z;q)∞ϕ22(a,cbc,az;q,bz).

This completes the proof.

## 5. Probabilistic Proofs of Some Formulas of *q*-Series

The *q*-binomial theorem is an important mathematical result which has been widely applied in the special functions, physics, quantum algebra, and quantum statistics. The *q*-binomial theorem was derived by Cauchy [23], Heine [22], and Jacobi [24] concerning the nonterminating form. There are many proofs of the *q*-binomial theorem to show the corresponding references; for example, a better and simpler proof, by using the method of the finite difference, was obtained by Askey (see [25]); a nice proof of the *q*-binomial theorem based on combinatorial considerations was given by Joichi and Stanton (see [26]). In 1847, Heine [22] derived a *q*-analogue of Gauss's summation formula which is important in *q*-series. Joichi and Stanton [26] gave a bijective proof of the *q*-Gauss summation formula based on combinatorial considerations. Rahman and Suslov [27] used the method of the first order linear difference equations to prove the *q*-Gauss summation formula. By analytic continuation, the terminating case, when *a* = *q* ^−*n*^, reduces to *q*-analogues of Vandermonde's formula. Bailey and Daum independently discovered the *q*-Kummer summation formula.

In this section we will introduce probabilistic proof of some formulas of *q*-series, for example, *q*-binomial theorem, *q*-Chu-Vandermonde, *q*-Gauss summation formula and *q*-Kummer summation formula, and so forth.

**Theorem 11 (see [16, page 488, Theorem 10.2.1] [17, page 354. II. 3])**

The *q*-binomial theorem is

(55) ϕ01(a−;q,z)=∑n=0∞(a;q)n(q;q)nzn=(az;q)∞(z;q)∞for  |z|<1, |q|<1.

**Proof**

Below we give two proofs of (55).

Setting *d* = *a* = 0 and replacing *b* and *c* by *a* and *b* in (14), we obtain

(56) E[1(aX,bX;q)∞]=1(ax,a,b;q)∞ϕ01(a−;q,bx).

Comparing (56) and (29) of Corollary 7, we have

(57) 1(ax,a,b;q)∞ϕ01(a−;q,bx)=(abx;q)∞(ax,bx,a,b;q)∞.

Then we obtain

(58) ϕ01(a−;q,bx)=(abx;q)∞(bx;q)∞.

Replacing *bx* by *z*, we can get

(59) ϕ01(a−;q,z)=(az;q)∞(z;q)∞;

that is,

(60) ϕ01(a−;q,z)=∑n=0∞(a;q)n(q;q)nzn=(az;q)∞(z;q)∞ for  |z|<1.

Another proof of the *q*-binomial theorem is as follows.

Setting *d* = *a* = 0 and *b* = *c* and replacing *b* by *a* in (14), we obtain

(61) E[1(aX;q)∞2]=1(ax,a,a;q)∞ϕ01(a−;q,ax).

Letting *a* = *b* = *c* = *d* or *a* = *b* and *d* = *c* in (14) of Theorem 3, we obtain

(62) E[1(aX;q)∞2]=(a,a2x;q)∞(ax,ax,a,a,a;q)∞ϕ23(a,a,1a2x,a;q,ax)=(a,a2x;q)∞(ax,ax,a,a,a;q)∞=(a2x;q)∞(ax,a;q)∞2.

Comparing (61) and (62) gives

(63) 1(ax,a,a;q)∞ϕ01(a−;q,ax)=(a2x;q)∞(ax,a;q)∞2.

Then we obtain

(64) ϕ01(a−;q,ax)=(a2x;q)∞(ax;q)∞.

Replacing *ax* by *z* gives

(65) ϕ01(a−;q,z)=(az;q)∞(z;q)∞;

that is,

(66) ϕ01(a−;q,z)=∑n=0∞(a;q)n(q;q)nzn=(az;q)∞(z;q)∞ for  |z|<1.

This proof is complete.

**Theorem 12 (see [17, page 354, II. 7])**

The *q*-Chu-Vandermonde sums are

(67) ϕ12(a,q−nc;q,cqna)=(c/a;q)n(c;q)n.

**Proof**

The below are two proofs of the *q*-Chu-Vandermonde.

(i) First proof: setting *d* = *a* = *b* and replacing *c* by *b* in (14), we have
  (68) E[1(aX,bX;q)∞]=(a2x;q)∞(ax,ax,a,b;q)∞ϕ12(a,aba2x;q,bx).

Replacing (*a*, *b*, *x*) by (*a*, *aq* ^*n*^, *c*/*a* ^2^) in (68), then we have

(69) P(Y=(ca2)nqk)   =pn,k(ca2;q) =∶(−c/a2)nqk((c/a2)n−1qk+1,(c/a2)nqk+1;q)∞(q,a2q/c,c/a2;q)∞,

where

(70) pn,k(ca2;q)>0,   ∑pn,k(ca2;q)=1,ca2<0,  0<q<1,  n=0,1,  k=0,1,2,….

Hence,

(71) E[1(aY,aqnY;q)∞] =(c;q)∞(c/a,c/a,a,aqn;q)∞ϕ12(a,q−nc;q,cqna).

By using the probability distribution *W*(*c*/*a* ^2^; *q*) and employing Andrews-Askey *q*-integral (11), now we calculate the expectation of the random variables 1/(*aY*,*aq* ^*n*^ *Y*;*q*)_*∞*_ as follows:

(72) E[1(aY,aqnY;q)∞]=∑n=01 ∑k=0∞(−c/a2)nqk((c/a2)n−1qk+1,(c/a2)nqk+1;q)∞(q,a2q/c,c/a2,a(c/a2)nqk,(aqn)(c/a2)nqk;q)∞=1(1−q)(q,a2q/c,c/a2;q)∞ ×((1−q)∑k=0∞(qk+1/(c/a2),qk+1;q)∞qk(aqk,(aqn)qk;q)∞−(ca2)(1−q)×∑k=0∞(qk+1,(c/a2)qk+1;q)∞qk(a(c/a2)qk,(aqn)(c/a2)qk;q)∞)=1(1−q)(q,a2q/c,c/a2;q)∞∫c/a21(qt/(c/a2),qt;q)∞(at,aqnt;q)∞dqt=1(1−q)(q,a2q/c,c/a2;q)∞(1−q)(q,a2q/c,c/a2,cqn;q)∞(c/a,cqn/a,a,aqn;q)∞=(cqn;q)∞(c/a,cqn/a,a,aqn;q)∞.

Comparing (71) and (72) gives

(73) (c;q)∞(c/a,c/a,a,aqn;q)∞ϕ12(a,q−nc;q,cqna)  =(cqn;q)∞(c/a,cqn/a,a,aqn;q)∞.

Then we obtain

(74) ϕ12(a,q−nc;q,cqna)  =(cqn;q)∞(c/a,cqn/a,a,aqn;q)∞(c/a,c/a,a,aqn;q)∞(c;q)∞  =(cqn;q)∞(c/a;q)∞(c;q)∞(cqn/a;q)∞  =(c/a;q)n(c;q)n,

which is just *q*-Vandermonde sums (67).

(ii) Second proof: replacing (*a*, *b*, *x*) by (*a*, *aq* ^*n*^, *c*/*a* ^2^) in (29), we have
  (75) $$\begin{array}{c} E\left[\frac{1}{{\left(aY,aq^{n}Y;q\right)}_{\infty }}\right]=\frac{{\left(cq^{n};q\right)}_{\infty }}{{\left(c/a,cq^{n}/a,a,aq^{n};q\right)}_{\infty }}. \end{array}$$

Comparing (71) and (75), we obtain

(76) (c;q)∞(c/a,c/a,a,aqn;q)∞ϕ12(a,q−nc;q,cqna)  =(cqn;q)∞(c/a,cqn/a,a,aqn;q)∞.

Hence,

(77) ϕ12(a,q−nc;q,cqna)=(cqn;q)∞(c/a;q)∞(c;q)∞(cqn/a;q)∞=(c/a;q)n(c;q)n,

which is just *q*-Vandermonde sums (67).

**Theorem 13 (see [16, page 522, Corollary 10.9.2] or [17, page 354, II. 8])**

The *q*-Gauss sum is

(78) ϕ12(a,bc;q,cab)=(c/a,c/b;q)∞(c,c/ab;q)∞.

**Proof**

Letting *d* = *a* = *b* and replacing *c* by *b* in (14), we obtain

(79) E[1(aX,bX;q)∞]=(a2x;q)∞(ax,ax,a,b;q)∞ϕ12(a,aba2x;q,bx).

Comparing (29) and (79) gives

(80) (a2x;q)∞(ax,ax,a,b;q)∞ϕ12(a,aba2x;q,bx)=(abx;q)∞(ax,bx,a,b;q)∞;

hence we get

(81) ϕ12(a,aba2x;q,bx)=(abx,ax;q)∞(a2x,bx;q)∞.

Replacing (*a*, *a*/*b*, *a* ^2^ *x*) by (*a*, *b*, *c*) in the above formula, we obtain

(82) ϕ12(a,bc;q,cab)=(c/a,c/b;q)∞(c,c/ab;q)∞,

which is just the *q*-Gauss sum (78).

**Theorem 14 (see [17, page 354, II. 9])**

The *q*-Kummer sum formula is

(83) ϕ12(a,baqb;q,−qb)=(−q;q)∞(  aq,aq2/b2;q2)∞(−q/b,aq/b;q)∞.

**Proof**

Letting *b* = 0 in (14) and then replacing *c* by *b*, we have

(84) E[(dX;q)∞(aX,bX;q)∞]=(d;q)∞(a,ax,b;q)∞ϕ12(a,dbd;q,bx).

Replacing (*a*, *b*, *d*, *x*) by (*a*, *aq*/*b* ^2^, *aq*/*b*, −*b*/*a*) in (84), we write

(85) P(Z=(−ba)nqk)  =pn,k(−ba;q)  =∶(b/a)nqk((−b/a)n−1qk+1,(−b/a)nqk+1;q)∞(q,−aq/b,−b/a;q)∞,

where

(86) pn,k(−ba;q)>0, ∑pn,k(−ba;q)=1,−ba<0,  0<q<1,  n=0,1,  k=0,1,2,….

Hence, we obtain

(87) E[((aq/b)Z;q)∞(aZ,(aq/b2)Z;q)∞]  =(aq/b;q)∞(a,−b,aq/b2;q)∞ϕ12(a,baqb;q,−qb).

By using the probability distribution *W*(−*b*/*a*; *q*) and Lemma 2, we calculate the expectation of the random variables ((*aq*/*b*)*Z*;*q*)_*∞*_/(*aZ*,(*aq*/*b* ^2^)*Z*;*q*)_*∞*_ as follows:

(88) E[((aq/b)Z;q)∞(aZ,(aq/b2)Z;q)∞]=E[((aq/b)Z;q)∞((aq/b2)Z,aZ;q)∞]=∑n=01 ∑k=0∞((ba)nqk((−ba)n−1qk+1,(−ba)nqk+1,(aqb)(−ba)nqk;q)∞×((q,−aqb,−ba,(aqb2)(−ba)nqk,a(−ba)nqk;q)∞)−1)=1(1−q)(q,−aq/b,−b/a;q)∞ ×((1−q)∑k=0∞(qk+1/(−b/a),qk+1,(aq/b)qk;q)∞qk((aq/b2)qk,aqk;q)∞−(−ba)(1−q)×∑k=0∞(qk+1,(−b/a)qk+1,(aq/b)(−b/a)qk;q)∞qk((aq/b2)(−b/a)qk,a(−b/a)qk;q)∞)=1(1−q)(q,−aq/b,−b/a;q)∞ ×∫−b/a1(qt/(−b/a),qt,(aq/b)t;q)∞((aq/b2)t,at;q)∞dqt=(1−q)(q,−aq/b,−b/a,aq/b;q)∞(1−q)(q,−aq/b,−b/a,−q/b,aq/b2,a;q)∞ϕ12 ×(aqb2,qbaqb;q,−b)=(aq/b;q)∞(−q/b,aq/b2,a;q)∞ϕ12(aqb2,qbaqb;q,−b).

Comparing (87) and (88), we have

(89) ϕ12(a,baqb;q,−qb)  =(a,−b,aq/b2;q)∞(aq/b;q)∞(aq/b;q)∞(−q/b,aq/b2,a;q)∞ϕ12   ×(aqb2,qbaqb;q,−b)  =(−b;q)∞(−q/b;q)∞ϕ12(aqb2,qbaqb;q,−b).

Using Heine's transformation and *q*-binomial theorem, we have

(90) ϕ12(a,baqb;q,−qb) =(−b;q)∞(−q/b;q)∞(a,−q;q)∞(aq/b,−b;q)∞ϕ12(−qb,qb−q;q,a) =(a,−q;q)∞(−q/b,aq/b;q)∞∑n=0∞(q2/b2;q2)n(q2;q2)nan =(a,−q;q)∞(−q/b,aq/b;q)∞(aq2/b2;q2)∞(a;q2)∞ =(−q;q)∞(aq,aq2/b2;q2)∞(−q/b,aq/b;q)∞.

Hence, we obtain (83).

**Theorem 15 (see [17, page 354, II. 10])**

Bailey's sum formula is

(91) ϕ22(a,qa−q,b;q,−b)=(ab,bq/a;q2)∞(b;q)∞.

**Proof**

By (19), we have

(92) E[(dX;q)∞(aX,bX;q)∞]=(d,abx;q)∞(a,ax,b,bx;q)∞ϕ22(a,babx,d;q,dx).

Replacing (*a*, *b*, *d*, *x*) by (*a*, *q*/*a*, −*q*, *b*/*q*) in (92) gives

(93) P(R=(bq)nqk)  =pn,k(bq;q)  =∶(−b/q)nqk((b/q)n−1qk+1,(b/q)nqk+1;q)∞(q,q2/b,b/q;q)∞,

where

(94) pn,k(bq;q)>0, ∑pn,k(bq;q)=1,bq<0,  0<q<1,  n=0,1,  k=0,1,2,….

Hence, we have

(95) E[(−qR;q)∞(aR,(q/a)R;q)∞]  =(−q,b;q)∞(a,ab/q,q/a,b/a;q)∞ϕ22(a,qab,−q;q,−b).

By using the probability distribution *W*(*b*/*q*; *q*) and Lemma 2, we calculate the expectation of the random variables (−*qR*; *q*)_*∞*_/(*aR*,(*q*/*a*)*R*;*q*)_*∞*_ as follows:

(96) E[(−qR;q)∞(aR,(q/a)R;q)∞]=∑n=01 ∑k=0∞((−bq)nqk((bq)n−1qk+1,(bq)nqk+1,(−q)(bq)nqk;q)∞×((q,q2b,bq,a(bq)nqk,(qa)(bq)nqk;q)∞)−1)=1(1−q)(q,q2/b,b/q;q)∞ ×((1−q)∑k=0∞(qk+1/(b/q),qk+1,(−q)qk;q)∞qk(aqk,(q/a)qk;q)∞−(bq)(1−q)×∑k=0∞(qk+1,(b/q)qk+1,(−q)(b/q)qk;q)∞qk(a(b/q)qk,(q/a)(b/q)qk;q)∞)=1(1−q)(q,q2/b,b/q;q)∞ ×∫b/q1(qt/(b/q),qt,−qt;q)∞(at,(q/a)t;q)∞dqt=1(1−q)(q,q2/b,b/q;q)∞(1−q)(q,q2/b,b/q,−q;q)∞(ab/q,a,q/a;q)∞ϕ12 ×(a,−a−q;q,ba)=(−q;q)∞(ab/q,a,q/a;q)∞ϕ12(a,−a−q;q,ba).

Comparing (95) and (96), we have

(97) ϕ22(a,qa−q,b;q,−b)=(b/a;q)∞(b;q)∞ϕ12(a,−a−q;q,ba)=(b/a;q)∞(b;q)∞∑n=0∞(a,−a;q)n(q,−q;q)n(ba)n=(b/a;q)∞(b;q)∞∑n=0∞(a2;q2)n(q2;q2)n(ba)n=(b/a;q)∞(b;q)∞(ab;q2)∞(b/a;q2)∞=(ab,bq/a;q2)∞(b;q)∞.

Hence, we get (91).

**Theorem 16 (see [17, page 354, II. 11])**

The Gauss sum formula is

(98) ϕ22(a2,b2abq1/2,−abq1/2;q,−q)=(a2q,b2q;q2)∞(q,a2b2q;q2)∞.

**Proof**

By (14), we have

(99) E[(dX;q)∞(aX,bX;q)∞] =(d,abx;q)∞(a,ax,b,bx;q)∞ϕ22(a,babx,d;q,dx).

Replacing (*a*, *b*, *d*, *x*) by (*a* ^2^, *b* ^2^, −*abq* ^1/2^, *q* ^1/2^/*ab*) in (99) gives

(100) P(S=(q1/2ab)nqk)=pn,k(q1/2ab;q)=∶(−q1/2/ab)nqk((q1/2/ab)n−1qk+1,(q1/2/ab)nqk+1;q)∞(q,abq1/2,q1/2/ab;q)∞,

where

(101) pn,k(q1/2ab;q)>0, ∑pn,k(q1/2ab;q)=1,q1/2ab<0,  0<q<1,  n=0,1,  k=0,1,2,….

Hence,

(102) E[(−abq1/2S;q)∞(a2S,b2S;q)∞]=(−abq1/2,abq1/2;q)∞(a2,aq1/2/b,b2,bq1/2/a;q)∞ϕ22 ×(a2,b2abq1/2,−abq1/2;q,−q).

By using the probability distribution *W*(*q* ^1/2^/*ab*; *q*) and employing Andrews-Askey *q*-integral (13) of Lemma 2, we calculate the expectation of the random variables (−*abq* ^1/2^ *S*; *q*)_*∞*_/(*a* ^2^ *S*,*b* ^2^ *S*;*q*)_*∞*_ as follows:

(103) E[(−abq1/2S;q)∞(a2S,b2S;q)∞]=∑n=01 ∑k=0∞((−q1/2ab)nqk((q1/2ab)n−1qk+1,(q1/2ab)nqk+1,(−abq1/2)(q1/2ab)nqk;q)∞×((q,abq1/2,q1/2ab,a2(q1/2ab)nqk,b2(q1/2ab)nqk;q)∞)−1)=1(1−q)(q,abq1/2,q1/2/ab;q)∞ ×((1−q)∑k=0∞(qk+1/(q1/2/ab),qk+1,(−abq1/2)qk;q)∞qk(a2qk,b2qk;q)∞−(q1/2ab)(1−q)×∑k=0∞(qk+1,(q1/2/ab)qk+1,(−abq1/2)(q1/2/ab)qk;q)∞qk(a2(q1/2/ab)qk,b2(q1/2/ab)qk;q)∞)=1(1−q)(q,abq1/2,q1/2/ab;q)∞ ×∫q1/2/ab1(qt/(q1/2/ab),qt,−abq1/2t;q)∞(a2t,b2t;q)∞dqt=(1−q)(q,abq1/2,q1/2/ab,−abq1/2;q)∞(1−q)(q,abq1/2,q1/2/ab,aq1/2/b,a2,b2;q)∞ϕ12 ×(a2,−aq1/2b−abq1/2;q,bq1/2a)=(−abq1/2;q)∞(aq1/2/b,a2,b2;q)∞ϕ12(a2,−aq1/2b−abq1/2;q,bq1/2a).

Comparing (102) and (103), we have

(104) ϕ22(a2,b2abq1/2,−abq1/2;q,−q)=(a2,aq1/2/b,b2,bq1/2/a;q)∞(−abq1/2,abq1/2;q)∞(−abq1/2;q)∞(aq1/2/b,a2,b2;q)∞ϕ12 ×(a2,−aq1/2b−abq1/2;q,bq1/2a)=(bq1/2/a;q)∞(abq1/2;q)∞ϕ12(a2,−aq1/2b−abq1/2;q,bq1/2a).

Using Heine's transformation formula, we have

(105) ϕ12(a2,−aq1/2b−abq1/2;q,bq1/2a)=(b2,−q;q)∞(−abq1/2,bq1/2/a;q)∞ϕ12(aq1/2b,−aq1/2b−q;q,b2).

Substituting (105) into (104) yields

(106) ϕ22(a2,b2abq1/2,−abq1/2;q,−q)=(bq1/2/a;q)∞(abq1/2;q)∞(b2,−q;q)∞(−abq1/2,bq1/2/a;q)∞ϕ12 ×(aq1/2b,−aq1/2b−q;q,b2)=(b2,−q;q)∞(abq1/2,−abq1/2;q)∞∑n=0∞(aq1/2/b,−aq1/2/b;q)n(q,−q;q)n(b2)n=(b2,−q;q)∞(abq1/2,−abq1/2;q)∞∑n=0∞(a2q/b2;q2)n(q2;q2)n(b2)n=(b2,−q;q)∞(abq1/2,−abq1/2;q)∞(a2q;q2)∞(b2;q2)∞.

Noting that

(107) $$\begin{array}{c} {\left(a^{2};q^{2}\right)}_{\infty }={\left(a;q\right)}_{\infty }{\left(-a;q\right)}_{\infty },\\ {\left(aq;q^{2}\right)}_{\infty }=\frac{{\left(a;q\right)}_{\infty }}{{\left(a;q^{2}\right)}_{\infty }}, \end{array}$$

we have

(108) $$\begin{array}{c} {\left(-q;q\right)}_{\infty }=\frac{{\left(-q;q\right)}_{\infty }{\left(q;q\right)}_{\infty }}{{\left(q;q\right)}_{\infty }}=\frac{{\left(q^{2};q^{2}\right)}_{\infty }}{{\left(q;q\right)}_{\infty }}=\frac{1}{{\left(q;q^{2}\right)}_{\infty }},\\ \frac{{\left(b^{2};q\right)}_{\infty }}{{\left(b^{2};q^{2}\right)}_{\infty }}={\left(b^{2}q;q^{2}\right)}_{\infty },\\ {\left(abq^{1/2};q\right)}_{\infty }{\left(-abq^{1/2};q\right)}_{\infty }={\left(a^{2}b^{2}q;q^{2}\right)}_{\infty }. \end{array}$$

Substituting (108) into (106) yields

(109) ϕ22(a2,b2abq1/2,−abq1/2;q,−q)=(a2q,b2q;q2)∞(q,a2b2q;q2)∞.

Hence, we obtain (98).

**Theorem 17 (see [17, page 354, II. 5])**

A sum formula of _1_ *ϕ*_1_ is

(110) ϕ11(ac;q,ca)=(c/a;q)∞(c;q)∞.

**Proof**

Letting *d* = *a* = *b* in (19) of Theorem 4, we obtain

(111) E[1(aX;q)∞]=(a2x;q)∞(ax,ax,a;q)∞ϕ11(aa2x;q,ax).

Letting *d* = *a* = *c* and *b* = 0 in (14) of Theorem 3 or setting *d* = *c* and *a* = 0 and replacing *b* by *a* in (14) of Theorem 3, we obtain

(112) $$\begin{array}{c} E\left[\frac{1}{{\left(aX;q\right)}_{\infty }}\right]=\frac{1}{{\left(ax,a;q\right)}_{\infty }}. \end{array}$$

Comparing (111) and (112) gives

(113) (a2x;q)∞(ax,ax,a;q)∞ϕ11(aa2x;q,ax)=1(ax,a;q)∞;

that is,

(114) ϕ11(aa2x;q,ax)=(ax;q)∞(a2x;q)∞.

Replacing (*a*, *a* ^2^ *x*) by (*a*, *c*), we get

(115) ϕ11(ac;q,ca)=(c/a;q)∞(c;q)∞.

This proof is complete.

**Theorem 18 (see [17, page 354, II. 1, II. 2])**

The two *q*-exponential functions are

(116) $$\begin{array}{c} e_{q}\left(z\right)=\overset{\infty }{\underset{n=0}{\sum }}\frac{z^{n}}{{\left(q;q\right)}_{n}}=\frac{1}{{\left(z;q\right)}_{\infty }}\;\text{for}\;\;\left|z\right|<1,\\ E_{q}\left(z\right)=\overset{\infty }{\underset{n=0}{\sum }}\frac{q^{\left(\begin{array}{c} n\\ 2 \end{array}\right)}z^{n}}{{\left(q;q\right)}_{n}}={\left(-z;q\right)}_{\infty }. \end{array}$$

**Proof**

Setting *a* = *b* = *d* = 0 and then replacing *c* by *a* in (14), we obtain

(117) E[1(aX;q)∞]=1(a;q)∞ϕ01(0−;q,ax).

Letting *d* = *a* = *c* and *b* = 0 in (14) or letting *d* = *c* and *a* = 0 and replacing *b* by *a* in (14), we obtain

(118) $$\begin{array}{c} E\left[\frac{1}{{\left(aX;q\right)}_{\infty }}\right]=\frac{1}{{\left(ax,a;q\right)}_{\infty }}. \end{array}$$

Comparing (117) and (118) gives

(119) 1(a;q)∞ϕ01(0−;q,ax)=1(ax,a;q)∞.

From the above formula, we obtain

(120) ϕ01(0−;q,ax)=1(ax;q)∞.

Replacing *ax* by *z* gives

(121) ϕ01(0−;q,z)=1(z;q)∞;

that is,

(122) $$\begin{array}{c} e_{q}\left(z\right)=\overset{\infty }{\underset{n=0}{\sum }}\frac{z^{n}}{{\left(q;q\right)}_{n}}=\frac{1}{{\left(z;q\right)}_{\infty }}\;\text{for}\;\;\left|z\right|<1. \end{array}$$

Similarly, setting *d* = *a* and *b* = 0 in (19) of Theorem 4, we obtain

(123) E[1]=1(ax;q)∞ϕ00(−−;q,ax).

And obviously letting *d* = *c* and *a* = *b* = 0 in (14) of Theorem 3, we obtain

(124) $$\begin{array}{c} E\left[1\right]=1. \end{array}$$

Comparing (123) and (124), we obtain

(125) 1(ax;q)∞ϕ00(−−;q,ax)=1.

Replacing *ax* by −*z*, we get

(126) ϕ00(−−;q,−z)=(−z;q)∞;

that is,

(127) $$\begin{array}{c} E_{q}\left(z\right)=\overset{\infty }{\underset{n=0}{\sum }}\frac{q^{\left(\begin{array}{c} n\\ 2 \end{array}\right)}z^{n}}{{\left(q;q\right)}_{n}}={\left(-z;q\right)}_{\infty }. \end{array}$$

**Remark 19**

In the present paper we obtain the part transformation and sum formulas of the *q*-series by applying the probabilistic method. We hope to find and construct another probability distribution in order to prove the transformation and sum formulas of the bilateral basic hypergeometric series, for example, Ramanujan and Bailey sum formulas and so forth.

## References

[1] Chang G, Xu C (2011). Generalization and probabilistic proof of a combinatorial identity. *The American Mathematical Monthly*.

[2] Chapman R (2005). A probabilistic proof of the Andrews-Gordon identities. *Discrete Mathematics*.

[3] Fulman J (2001). A probabilistic proof of the Rogers-Ramanujan identities. *Bulletin of the London Mathematical Society*.

[4] Kadell KWJ (1987). A probabilistic proof of Ramanujan's _1_
*ψ*
_1_ sum. *SIAM Journal on Mathematical Analysis*.

[5] Lee PA (1997). Probability distribution and a self-reciprocal relation involving confluent hypergeometric functions. *Journal of the Franklin Institute*.

[6] Ong SH (1999). Probabilistic proof of a Hankel transform of Laguerre polynomials. *Journal of the Franklin Institute*.

[7] Pascu MN (2005). A probabilistic proof of the fundamental theorem of algebra. *Proceedings of the American Mathematical Society*.

[8] Rosalsky A (2007). A simple and probabilistic proof of the binomial theorem. *The American Statistician*.

[9] Ross SM (2010). *A First Course in Probability*.

[10] Srivastava HM, Vignat C (2012). Probabilistic proofs of some relationships between the Bernoulli and Euler polynomials. *European Journal of Pure and Applied Mathematics*.

[11] Miller SJ (2008). A probabilistic proof of Wallis's formula for *π*. *The American Mathematical Monthly*.

[12] Sun P (2007). Moment representation of Bernoulli polynomial, Euler polynomial and Gegenbauer polynomials. *Statistics & Probability Letters*.

[13] Wang M (2011). An expectation formula with applications. *Journal of Mathematical Analysis and Applications*.

[14] Wang M (2010). A new probability distribution with applications. *Pacific Journal of Mathematics*.

[15] Xia A (1999). A probabilistic proof of Stein's factors. *Journal of Applied Probability*.

[16] Andrews GE, Askey R, Roy R (1999). *Special Functions*.

[17] Gasper G, Rahman M (2004). *Basic Hypergeometric Series*.

[18] Jackson FH (1910). On *q*-definite integrals. *The Quarterly Journal of Pure and Applied Mathematics*.

[19] Andrews GE, Askey R (1981). Another *q*-extension of the beta function. *Proceedings of the American Mathematical Society*.

[20] Liu DF, Luo QM An extension for Andrews-Askey integral formula.

[21] Rahman M, Verma A (1986). A *q*-integral representation of Rogers' *q*-ultraspherical polynomials and some applications. *Constructive Approximation*.

[22] Heine E (1847). Untersuchungen über die Reihe. *Journal für die reine und angewandte Mathematik*.

[23] Cauchy A-L (1843). Mémoire sur les fonctions dont plusieurs valeurs sont liées entre elles par une équation linéaire, et sur diverses transformations de produits composés d’un nombre indéfini de facteurs. *Comptes Rendus de l'Académie des Sciences*.

[24] Jacobi CGJ (1846). Über einiger der Binomialreihe analoge Reihen. *Journal für die reine und angewandte Mathematik*.

[25] Askey R (1980). Ramanujan's extensions of the gamma and beta functions. *The American Mathematical Monthly*.

[26] Joichi JT, Stanton D (1987). Bijective proofs of basic hypergeometric series identities. *Pacific Journal of Mathematics*.

[27] Rahman M, Suslov SK (1996). A unified approach to the summation and integration formulas for *q*-hypergeometric functions I. *Journal of Statistical Planning and Inference*.

